# Spurious oscillations reduction in transient diffusion and wave propagation problems discretized with the Finite Element Method

**DOI:** 10.1038/s41598-022-23185-x

**Published:** 2022-11-07

**Authors:** Augusto Badke Neto, Webe João Mansur, Walnório Graça Ferreira

**Affiliations:** 1 COPPE/Department of Civil Engineering, Federal University of Rio de Janeiro, Rio de Janeiro, Brazil; 2 Department of Civil Engineering, Federal University of Espírito Santo, Vitória, Brazil; 3 LAMEMO-Federal University of Rio de Janeiro, Rio de Janeiro, Brazil

**Keywords:** Engineering, Civil engineering

## Abstract

This work presents a new method, the SOR_FEM, spurious oscillation reduction-FEM, that uses analytical solutions of simple problems to find numerical solutions of more complex problems to reduce the spurious oscillations that occur due to solution discontinuity. With the proposed method it is possible to find numerical solutions without spurious oscillations of problems submitted to impulsive point sources without using any approximation for the Dirac delta function. To validate the proposed method, linear problems of heat diffusion and wave propagation in homogeneous and heterogeneous media are solved with the Finite Element Method (FEM).

## Introduction

Many physical phenomena studied in engineering such as heat transfer, fluid mechanics, solid mechanics, wave propagation, etc., can be mathematically represented by partial differential equations. To obtain the solutions of these equations, either analytical or numerical methods can be applied. Analytical solution methods provide exact solutions, but their application is restricted to simple problems^1^. Numerical methods provide approximate solutions; however, these methods can be applied to non-homogeneous, non-linear problems with complex boundary conditions and irregular geometry^1^.

The Finite Element Method (FEM) is a numerical method widely used in engineering analysis for solving complex mathematical problems^2^. However, it is difficult for the FEM to approximate solutions with discontinuity^3^. Transient wave propagation problems solutions which may exhibit spurious oscillations around a discontinuity point, related to the Gibbs phenomenon, may also exhibit amplitude decay and numerical period elongation, which makes the numerical wave propagation velocity different from the physical velocity resulting in dissipation and dispersion errors^4–7^.

There are distinct methods in the literature that aim to reduce the spurious oscillations that occur in the numerical solution of wave propagation problems. Various research works have been conducted to develop time integration methods with controlled numerical damping to dissipate numerical, thus spurious, high frequencies^8–19^. Variations of the discontinuous Galerkin method have been studied, in which the method is applied in the time and space discretizations to reduce spurious oscillations around discontinuities^20–22^. Other authors proposed modifications for the spatial integration rule used to obtain the mass and stiffness matrices^6,7,23^. Ham and Bathe developed an enriched finite element method that combines the advantages of the spectral method, which uses harmonic functions as base functions, with the advantages of the finite element method to solve wave propagation problems^4^. Studies show that it is possible to use a post-processing method to filter the noise in the solution and, thus, errors of the numerical solutions^3,24,25^.

Solutions discontinuities can occur when discontinuous functions, such as the Dirac delta function, are used as the source term in partial differential equations. The Dirac delta function is used to represent the effect of point sources or instantaneous impulses in partial differential equations, as in the case of concentrated loads, moving loads, impact loads, explosions, etc. In problems in which the Dirac delta function is used as the source term in the partial differential equation, published research works show that it may be necessary to adopt a smooth approximation for this function^26–30^.

This work presents a method that uses the analytical solution of a simple problem to find the numerical solution of a more complex problem to reduce the spurious oscillations that occur due to the solution discontinuity. In the proposed method worked out here, whose preliminary ideas were presented in^31^, algebraic manipulations are performed on the partial differential equation to be solved to replace the source term by an auxiliary analytical solution of a known problem. With the proposed method it is possible to find numerical solutions without spurious oscillations of problems submitted to impulsive point sources without using an approximation for the Dirac delta function. To validate the proposed method, linear problems of heat diffusion and wave propagation in homogeneous and heterogeneous media are solved with the finite element method.

It is important to remark that no numerical diffusion is performed by the proposed method; however, sometimes a local adaptation of the auxiliary solution may be necessary. Herein local adaptations of the auxiliary solutions were only necessary for the wave propagation problems.

## Methodology

### Heat diffusion

The linear heat diffusion problem in heterogeneous two-dimensional media is defined by the following differential equation:

1 $$\rho \left(x,y\right)c\left(x,y\right)\frac{\partial u}{\partial t}-\frac{\partial }{\partial x}\left[\lambda \left(x,y\right)\frac{\partial u}{\partial x}\right]-\frac{\partial }{\partial y}\left[\lambda \left(x,y\right)\frac{\partial u}{\partial y}\right]=f\left(x,y,t\right),$$

where $u=u(x,y,t)$ is the media temperature at point $(x,y)$ at time $t$, $f(x,y,t)$ is the source term, $c$ is the specific heat capacity, $\rho$ is the density and $\lambda$ is the thermal conductivity.

In this paper ${u}^{*}(x,y,t)$ denotes known analytical solution. For linear heat diffusion problems in two-dimensional homogeneous media and source term equal to that given by Eq. (1), u* can be obtained solving Eq. (2) below:

2 $${\rho }_{0}{c}_{0}\frac{\partial {u}^{*}}{\partial t}-\frac{\partial }{\partial x}\left({\lambda }_{0}\frac{\partial {u}^{*}}{\partial x}\right)-\frac{\partial }{\partial y}\left({\lambda }_{0}\frac{\partial {u}^{*}}{\partial y}\right)=f\left(x,y,t\right),$$

where ${\rho }_{0}$, ${c}_{0}$ and ${\lambda }_{0}$ are the properties of the homogeneous media.

The proposed method replaces the source term indicated in Eq. (1) by smoothers source terms given by the left-hand-side of Eq. (2), which are more amenable for numerical treatment by the FEM. Equalizing left-hand-sides of Eqs. (1) and (2), subsequently subtracting $\rho \left(x,y\right)c\left(x,y\right)\partial {u}^{*}/\partial t,$ adding $\partial /\partial x[\lambda \left(x,y\right)\partial {u}^{*}/\partial x]$ and $\partial /\partial y[\lambda \left(x,y\right)\partial {u}^{*}/\partial y]$ to both sides of the resulting equation one obtains:

3 $$\rho \left(x,y\right)c\left(x,y\right)\frac{\partial u}{\partial t}-\rho \left(x,y\right)c\left(x,y\right)\frac{\partial {u}^{*}}{\partial t}-\frac{\partial }{\partial x}\left[\lambda \left(x,y\right)\frac{\partial u}{\partial x}\right]+\frac{\partial }{\partial x}\left[\lambda \left(x,y\right)\frac{\partial {u}^{*}}{\partial x}\right]-\frac{\partial }{\partial y}\left[\lambda \left(x,y\right)\frac{\partial u}{\partial y}\right]+\frac{\partial }{\partial y}\left[\lambda \left(x,y\right)\frac{\partial {u}^{*}}{\partial y}\right]={\rho }_{0}{c}_{0}\frac{\partial {u}^{*}}{\partial t}-\rho \left(x,y\right)c\left(x,y\right)\frac{\partial {u}^{*}}{\partial t}-\frac{\partial }{\partial x}\left({\lambda }_{0}\frac{\partial {u}^{*}}{\partial x}\right)+\frac{\partial }{\partial x}\left[\lambda \left(x,y\right)\frac{\partial {u}^{*}}{\partial x}\right]-\frac{\partial }{\partial y}\left({\lambda }_{0}\frac{\partial {u}^{*}}{\partial y}\right)+\frac{\partial }{\partial y}\left[\lambda \left(x,y\right)\frac{\partial {u}^{*}}{\partial y}\right].$$

Considering $\Delta u=u-{u}^{*}$, $\Delta c=\rho \left(x,y\right)c\left(x,y\right)-{\rho }_{0}{c}_{0}$ and $\Delta \lambda =\lambda \left(x,y\right)-{\lambda }_{0}$ one obtains:

4 $$\rho \left(x,y\right)c\left(x,y\right)\frac{\partial\Delta u}{\partial t}-\frac{\partial }{\partial x}\left[\lambda \left(x,y\right)\frac{\partial\Delta u}{\partial x}\right]-\frac{\partial }{\partial y}\left[\lambda \left(x,y\right)\frac{\partial\Delta u}{\partial y}\right]=-\Delta c\frac{\partial {u}^{*}}{\partial t}+\frac{\partial }{\partial x}\left(\Delta \lambda \frac{\partial {u}^{*}}{\partial x}\right)+\frac{\partial }{\partial y}\left(\Delta \lambda \frac{\partial {u}^{*}}{\partial y}\right).$$

Equation (4) can be discretized in space by weighted residuals^32^ as indicated by Eq. (5),

5 $${\int }_{\Omega }{R}_{\Omega }{W}_{i}d\Omega +{\int }_{\Gamma }{R}_{\Gamma }{\overline{W}}_{i}d\Gamma =0,$$

where $\Omega$ is an open bounded set in ${\mathbb{R}}^{2}$ with Lipschitz boundary denoted by $\Gamma$, ${R}_{\Omega }$ is the residual in the $\Omega$ domain, ${R}_{\Gamma }$ is the residual on the $\Gamma$ boundary, ${W}_{i}$ and ${\overline{W}}_{i}$ are weighting functions.

The Galerkin approach leads to the following weak FEM matrix expression:

6 $${\varvec{C}}{\varvec{\Delta}}\dot{{\varvec{u}}}+{\varvec{K}}{\varvec{\Delta}}{\varvec{u}}={\varvec{f}}-{\varvec{\Delta}}{\varvec{C}}{\dot{{\varvec{u}}}}^{\boldsymbol{*}}-{\varvec{\Delta}}{\varvec{K}}{{\varvec{u}}}^{\boldsymbol{*}},$$

where ${\varvec{\Delta}}{\varvec{C}}={\varvec{C}}-{{\varvec{C}}}_{0}$,${\varvec{\Delta}}{\varvec{K}}={\varvec{K}}-{{\varvec{K}}}_{0}$, ${\varvec{C}}$ is the thermal capacity matrix, ${\varvec{K}}$ is the thermal conductivity matrix and ${\varvec{f}}$ is the heat flux vector, given by:

7 $${C}_{\mathrm{i},\mathrm{j}}={\int }_{\Omega }\rho \left(x,y\right)c(x,y){N}_{i}{N}_{j}d\Omega,$$

8 $${{C}_{0}}_{\mathrm{i},\mathrm{j}}={\rho }_{0}{c}_{0}{\int }_{\Omega }{N}_{i}{N}_{j}d\Omega ,$$

9 $${K}_{\mathrm{i},\mathrm{j}}={\int }_{\Omega }\lambda (x,y)\left(\frac{{\partial N}_{i}}{\partial x}\frac{{\partial N}_{j}}{\partial x}+\frac{{\partial N}_{i}}{\partial y}\frac{{\partial N}_{j}}{\partial y}\right)d\Omega ,$$

10 $${{K}_{0}}_{\mathrm{i},\mathrm{j}}={\uplambda }_{0}{\int }_{\Omega }\left(\frac{{\partial N}_{i}}{\partial x}\frac{{\partial N}_{j}}{\partial x}+\frac{{\partial N}_{i}}{\partial y}\frac{{\partial N}_{j}}{\partial y}\right)d\Omega,$$

11 $${f}_{\mathrm{i}}={\int }_{\Gamma }\lambda \left(x,y\right)\left(\frac{\partial\Delta u}{\partial x}{n}_{x}+\frac{\partial\Delta u}{\partial y}{n}_{y}\right){N}_{i}d\Gamma +{\int }_{\Gamma }\Delta \lambda \left(\frac{\partial {\mathrm{u}}^{*}}{\partial x}{n}_{x}+\frac{\partial {\mathrm{u}}^{*}}{\partial y}{n}_{y}\right){N}_{i}d\Gamma .$$

Note that $N$ is shape function, and also weighting function as the Galerkin approach is used here.

The finite difference method can be applied in the time discretization of Eq. (6) as indicated below:

$$\boldsymbol{\Delta }{\dot{{\varvec{u}}}}_{{t}_{i}+\theta \Delta t}=\frac{\boldsymbol{\Delta }{{\varvec{u}}}_{{t}_{i}+\Delta t}-\boldsymbol{\Delta }{{\varvec{u}}}_{{t}_{i}}}{\Delta t},$$

12 $$\boldsymbol{\Delta }{{\varvec{u}}}_{{t}_{i}+\theta \Delta t}=\left(1-\theta \right)\boldsymbol{\Delta }{{\varvec{u}}}_{{t}_{i}}+\theta \boldsymbol{\Delta }{{\varvec{u}}}_{{t}_{i}+\Delta t}.$$

The solution for the time ${t}_{i}+\Delta t$ is obtained by considering Eq. (6) at time ${t}_{i}+\theta\Delta t$, then $\boldsymbol{\Delta }{\dot{{\varvec{u}}}}_{{t}_{i}+\theta \Delta t}$ and $\boldsymbol{\Delta }{{\varvec{u}}}_{{t}_{i}+\theta \Delta t}$ given above can be substituted into Eq. (6) to obtain:

13 $$\left(\frac{{\varvec{C}}}{\Delta t}+\theta {\varvec{K}}\right)\boldsymbol{\Delta }{{\varvec{u}}}_{{t}_{i}+\Delta t}=\left[\frac{\mathbf{C}}{\Delta t}-\left(1-\theta \right){\varvec{K}}\right]\boldsymbol{\Delta }{{\varvec{u}}}_{{t}_{i}}+{{\varvec{f}}}_{{t}_{i}+\theta \Delta t}-\boldsymbol{\Delta }{\varvec{C}}{\dot{{\varvec{u}}}}_{{t}_{i}+\theta \Delta t}^{\boldsymbol{*}}-\boldsymbol{\Delta }{\varvec{K}}{{\varvec{u}}}_{{t}_{i}+\theta \Delta t}^{\boldsymbol{*}}.$$

The parameter $\theta =0.5$ used here, leads to the Crank–Nicholson scheme. Equation (13) gives rise to an implicit algorithm, unconditionally stable. The answer of the problem is found by computing at each discrete time ${{\varvec{u}}}_{{t}_{i}+\Delta t}={\varvec{\Delta}}{{\varvec{u}}}_{{t}_{i}+\Delta t}+{{\varvec{u}}}_{{t}_{i}+\Delta t}^{\boldsymbol{*}}$.

### Wave propagation

The linear wave propagation problem in heterogeneous two-dimensional media is defined by the following differential equation:

14 $$\rho \left(x,y\right)A\frac{{\partial }^{2}u}{\partial {t}^{2}}-\frac{\partial }{\partial x}\left[E\left(x,y\right)A\frac{\partial u}{\partial x}\right]-\frac{\partial }{\partial y}\left[E\left(x,y\right)A\frac{\partial u}{\partial y}\right]=f\left(x,y,t\right),$$

where $u=u(x,y,t)$ is the displacement at the point $(x,y)$ at time $t$, $f(x,y,t)$ is the source term, $\rho$ is the density, $E$ is the Young modulus and $A$ is the thickness.

The proposed method for wave propagation problems can be established following a procedure like that shown in the previous subsection, resulting in the following expression:

15 $$\rho \left(x,y\right)A\frac{{\partial }^{2}\Delta u}{\partial {t}^{2}}-\frac{\partial }{\partial x}\left[E\left(x,y\right)A\frac{\partial\Delta u}{\partial x}\right]-\frac{\partial }{\partial y}\left[E\left(x,y\right)A\frac{\partial\Delta u}{\partial y}\right]=-\Delta \rho A\frac{{\partial }^{2}{u}^{*}}{\partial {t}^{2}}+\frac{\partial }{\partial x}\left(\Delta EA\frac{\partial {u}^{*}}{\partial x}\right)+\frac{\partial }{\partial y}\left(\Delta EA\frac{\partial {u}^{*}}{\partial y}\right),$$

where $$\Delta u=u-{u}^{*}$$, $$\Delta \rho =\rho \left(x,y\right)-{\rho }_{0}$$ and $$\Delta E=E\left(x,y\right)-{E}_{0}$$.

Applying weighted residuals, followed by the usual Galerkin procedure and the classical FEM space discretization the following matrix expression results:

16 $${\varvec{M}}{\varvec{\Delta}}\ddot{{\varvec{u}}}+{\varvec{K}}{\varvec{\Delta}}{\varvec{u}}={\varvec{f}}-{\varvec{\Delta}}{\varvec{M}}{\ddot{{\varvec{u}}}}^{\boldsymbol{*}}-{\varvec{\Delta}}{\varvec{K}}{{\varvec{u}}}^{\boldsymbol{*}},$$

where ${\varvec{\Delta}}{\varvec{M}}={\varvec{M}}-{{\varvec{M}}}_{0}$,${\varvec{\Delta}}{\varvec{K}}={\varvec{K}}-{{\varvec{K}}}_{0}$, ${\varvec{M}}$ is the mass matrix, ${\varvec{K}}$ is the stiffness matrix and ${\varvec{f}}$ is the force vector, given by:

17 $${M}_{\mathrm{i},\mathrm{j}}={\int }_{\Omega }\rho \left(x,y\right)A{N}_{i}{N}_{j}d\Omega,$$

18 $${{M}_{0}}_{\mathrm{i},\mathrm{j}}={\rho }_{0}A{\int }_{\Omega }{N}_{i}{N}_{j}d\Omega,$$

19 $${K}_{\mathrm{i},\mathrm{j}}={\int }_{\Omega }E(x,y)A\left(\frac{{\partial N}_{i}}{\partial x}\frac{{\partial N}_{j}}{\partial x}+\frac{{\partial N}_{i}}{\partial y}\frac{{\partial N}_{j}}{\partial y}\right)d\Omega ,$$

20 $${{K}_{0}}_{\mathrm{i},\mathrm{j}}={\mathrm{E}}_{0}A{\int }_{\Omega }\left(\frac{{\partial N}_{i}}{\partial x}\frac{{\partial N}_{j}}{\partial x}+\frac{{\partial N}_{i}}{\partial y}\frac{{\partial N}_{j}}{\partial y}\right)d\Omega,$$

21 $${f}_{\mathrm{i}}={\int }_{\Gamma }E\left(x,y\right)A\left(\frac{\partial\Delta u}{\partial x}{n}_{x}+\frac{\partial\Delta u}{\partial y}{n}_{y}\right){N}_{i}d\Gamma +{\int }_{\Gamma }\Delta EA\left(\frac{\partial {\mathrm{u}}^{*}}{\partial x}{n}_{x}+\frac{\partial {\mathrm{u}}^{*}}{\partial y}{n}_{y}\right){N}_{i}d\Gamma .$$

The Newmark method indicated below

22 $${\varvec{\Delta}}{{\varvec{u}}}_{{t}_{i}+\Delta t}={\varvec{\Delta}}{{\varvec{u}}}_{{t}_{i}}+{\Delta }_{t}{\varvec{\Delta}}{\dot{{\varvec{u}}}}_{{t}_{i}}+\frac{\Delta {t}^{2}}{2}\left[(1-2\beta ){\varvec{\Delta}}{\ddot{{\varvec{u}}}}_{{t}_{i}}+2\beta{\varvec{\Delta}}{\ddot{{\varvec{u}}}}_{{t}_{i}+\Delta t}\right],$$

23 $${\varvec{\Delta}}{\dot{{\varvec{u}}}}_{{t}_{i}+\Delta t}={\varvec{\Delta}}{\dot{{\varvec{u}}}}_{{t}_{i}}+\Delta t\left[(1-\gamma ){\varvec{\Delta}}{\ddot{{\varvec{u}}}}_{{t}_{i}}+\gamma{\varvec{\Delta}}{\ddot{{\varvec{u}}}}_{{t}_{i}+\Delta t}\right],$$

applied to discretize Eq. (16) in time gives:

24 $$\left(\frac{\gamma }{\beta\Delta {t}^{2}}{\varvec{M}}+{\varvec{K}}\right){\varvec{\Delta}}{{\varvec{u}}}_{{t}_{i}+\Delta t}={{\varvec{f}}}_{{t}_{i}+\mathrm{\Delta t}}-{\varvec{\Delta}}{\varvec{M}}{\ddot{{\varvec{u}}}}_{{t}_{i}+\Delta t}^{\boldsymbol{*}}-{\varvec{\Delta}}{\varvec{K}}{{\varvec{u}}}_{{t}_{i}+\Delta t}^{\boldsymbol{*}}+{\varvec{M}}\left[\frac{1}{\beta\Delta {t}^{2}}{\varvec{\Delta}}{{\varvec{u}}}_{{t}_{i}}+\left(\frac{1}{\beta\Delta t}\right){\varvec{\Delta}}{\dot{{\varvec{u}}}}_{{t}_{i}}+\left(\frac{1}{2\beta }-1\right){\varvec{\Delta}}{\ddot{{\varvec{u}}}}_{{t}_{i}}\right].$$

The parameters $\beta =\frac{1}{4}$ and $\gamma =\frac{1}{2}$ used in Eqs. (22) and (23) led to the implicit approach indicated by Eq. (24). The answer to the problem is found by computing at each discrete time ${{\varvec{u}}}_{{t}_{i}+\Delta t}={\varvec{\Delta}}{{\varvec{u}}}_{{t}_{i}+\Delta t}+{{\varvec{u}}}_{{t}_{i}+\Delta t}^{\boldsymbol{*}}$.

## Validation

### Example 1

**Figure 1:**
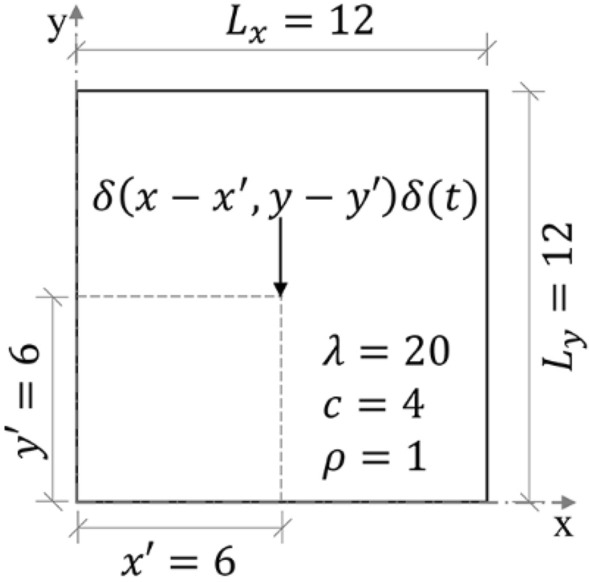
The problem solved in example 1.

In this problem, one finds the temperature resulting from the application of an impulsive point source $\delta \left(x-{x}{^{\prime}},y-y{^{\prime}}\right)\delta \left(t\right)$ in a two-dimensional square domain, as illustrated in Fig. 1, with Dirichlet boundary condition ${u}_{{\Gamma }_{D}}=0$ on the whole boundary. The initial condition adopted is $u(x,y,0)=0$.

According to Carslaw and Jaeger^33^, the analytical solution for this example is given by:

25 $${u}_{an}(x,y,t)=\frac{4}{{L}_{x}{L}_{y}}\sum_{n=1}^{\infty }\sum_{m=1}^{\infty }{e}^{-\kappa {\pi }^{2}t\left(\frac{{m}^{2}}{{L}_{x}{L}_{y}}+\frac{{n}^{2}}{{L}_{x}{L}_{y}}\right)}\mathrm{sin}\left(\frac{m\pi x}{{L}_{x}}\right)\mathrm{sin}\left(\frac{m\pi x{^{\prime}}}{{L}_{x}}\right)\mathrm{sin}\left(\frac{n\pi y}{{L}_{y}}\right)\mathrm{sin}\left(\frac{n\pi y{^{\prime}}}{{L}_{y}}\right),$$

where $\kappa$ is the thermal diffusivity defined by $\kappa =\frac{\lambda }{\rho c}$, ${x}^{{\prime}}=6$ and ${y}^{{\prime}}=6$ are the source point coordinates and ${L}_{x}$ and ${L}_{y}$ are the domain sides.

Since the domain under analysis is homogeneous the terms ${\varvec{\Delta}}\mathbf{C}$ and ${\varvec{\Delta}}\mathbf{K}$ of the Eq. (13) are null, so the governing equation to be solved for $\theta =0.5$ is given by:

26 $$\left(\frac{{\varvec{C}}}{\mathrm{\Delta t}}+\frac{{\varvec{K}}}{2}\right)\boldsymbol{\Delta }{{\varvec{u}}}_{{t}_{i}+\Delta t}=\left(\frac{\mathbf{C}}{\Delta t}-\frac{{\varvec{K}}}{2}\right)\boldsymbol{\Delta }{{\varvec{u}}}_{{t}_{i}}.$$

To apply the developed method, it was used the analytical solution of an infinite two-dimensional homogeneous domain problem submitted to the same source term illustrated in Fig. 1. According to Carslaw and Jaeger^33^, this analytical solution, denoted as ${u}^{*}$ in Eq. (3), is given by:

27 $${u}^{*}=\frac{1}{4\pi \kappa t}{e}^{-\frac{{\left(x-{x}{^{\prime}}\right)}^{2}+{\left(y-{y}{^{\prime}}\right)}^{2}}{4\kappa t}}.$$

Boundary conditions must satisfy over the $\Gamma$ boundary an equation like $au\left(x,y,t\right)+\mathrm{b}\partial \mathrm{u}(\mathrm{x},\mathrm{y},\mathrm{t})/\partial \mathrm{n} =c(x,y,t)$, $x,y\in\Gamma$, where $\mathrm{b}=0$ gives the Dirichlet boundary condition and $a=0$ gives the Neumann boundary condition. One can obey the boundary condition of the original problem if the equation to be solved, for instance Eq. (26), or else any other one obeys: $a\Delta u\left(x,y,t\right)+\mathrm{b}\partial \mathrm{\Delta u}(\mathrm{x},\mathrm{y},\mathrm{t})/\partial \mathrm{n} =c(x,y,t)-a{u}^{*} \left(x,y,t\right)-\mathrm{b}\partial {\mathrm{u}}^{*}(\mathrm{x},\mathrm{y},\mathrm{t})/\partial \mathrm{n})$, $x,y\in\Gamma$. Thus, the Dirichlet boundary condition to solve Eq. (26) is defined by:

28 $$\Delta {u}_{{\Gamma }_{D} }={u}_{{\Gamma }_{D}}-{u}^{*}\left({x}_{{\Gamma }_{D}},{y}_{{\Gamma }_{D}},t\right)=0-{u}^{*}\left({x}_{{\Gamma }_{D}},{y}_{{\Gamma }_{D}},t\right),$$

where ${x}_{{\Gamma }_{D}}$ and ${y}_{{\Gamma }_{D}}$ are boundary coordinates of the domain illustrated in Fig. 1.

**Figure 2:**
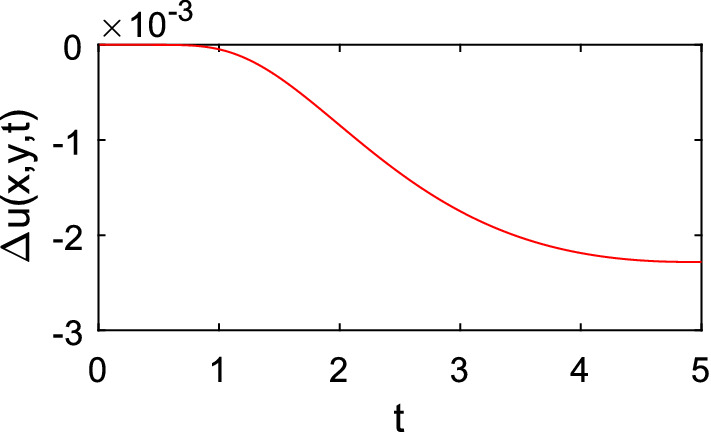
Time history of $\Delta u$ at the point $(\mathrm{6,6})$.

The proposed method, SOR_FEM (spurious oscillation reduction-FEM), employed a uniform mesh composed of 3600 quadrilateral bilinear elements with four nodes. The element sides were equal to 0.2. The time-step used was $\Delta t=0.01$. At the end of each time-step the proposed method solution was computed by $u=\Delta u+{u}^{*}$. Figure 2 shows the time history of $\Delta u$ at the source application point.

Since $\Delta u=u-{u}^{*}$, the solution of $\Delta u$ results on the correction applied on the ${u}^{*}$ values to match the $u$ values. On the first time-steps no relevant correction on the ${u}^{*}$ solution is needed since the value on the boundary given by Eq. (28) is close to 0 and, thus is $\Delta u$. In the classical methods on the first time steps the solution of $u$ is concentrated around the source application point while in the proposed method the solution is smooth, as shown in Fig. 2, thus the latter is more amenable for numerical treatment by the Finite Element Method.

The proposed method was compared to a conventional method which consists of discretizing Eq. (1) in space by Galerkin approach, which results in the following matrix equation:

29 $${\varvec{C}}\dot{{\varvec{u}}}+{\varvec{K}}{\varvec{u}}={\varvec{g}},$$

where ${\varvec{g}}$ has only one nonnull element ${g}_{\mathrm{i}}={\int }_{\Omega }\delta (x-{x}{^{\prime}},y-y{^{\prime}})\delta (t){N}_{i}d\Omega$, where “i” indicates node number with coordinates $(\mathrm{6,6})$.

The Eq. (29) was discretized in time using two different methods, the Cranck-Nicolson and the Implicit Euler. The application of the Implicit Euler method to Eq. (29) results in the following expression:

30 $$\left({\varvec{C}}+\Delta t{\varvec{K}}\right){{\varvec{u}}}_{{t}_{i}+\Delta t}={\varvec{C}}{{\varvec{u}}}_{{t}_{i}}+{\int }_{{t}_{i}}^{{t}_{i}+\Delta t}{\varvec{g}}dt.$$

The answer obtained through Eq. (30) was called G-IE, this response was found using the same mesh and time discretization defined above for the proposed method. The implicit Euler was chosen because of its low cost and enhanced stability, although being first order accurate^2^.

The application of the Cranck-Nicolson method in Eq. (29) results in the following expression:

31 $$\left(\frac{{\varvec{C}}}{\Delta t}+\frac{{\varvec{K}}}{2}\right){{\varvec{u}}}_{{t}_{i}+\Delta t}=\left(\frac{{\varvec{C}}}{\Delta t}-\frac{{\varvec{K}}}{2}\right){{\varvec{u}}}_{{t}_{i}}.$$

To represent the effect of the source $\delta (x-{x}{^{\prime}},y-y{^{\prime}})\delta (t)$ in Eq. (31) it was used the method described by Lardner and Song^34^ which consists of using $u\left(\mathrm{6,6},0\right)=K/(\Delta x\Delta y)$ as the initial condition, where $K$ is the energy released by the pulse, $\Delta x$ and $\Delta y$ are respectively the lengths of the element sides in $x$ and $y$ directions. The answer obtained through Eq. (31) was called G-CN, the answer was found considering $K=1$ and using the same mesh and time discretization defined above for the proposed method. The Cranck-Nicolson method was chosen because it is second-order accurate and may result in responses with spurious oscillations when applied to problems submitted to impulsive point sources. Since the Cranck-Nicolson method was also applied in time discretization of the SOR_FEM, it is possible to show that the SOR_FEM results in a more stable solution.

**Figure 3:**
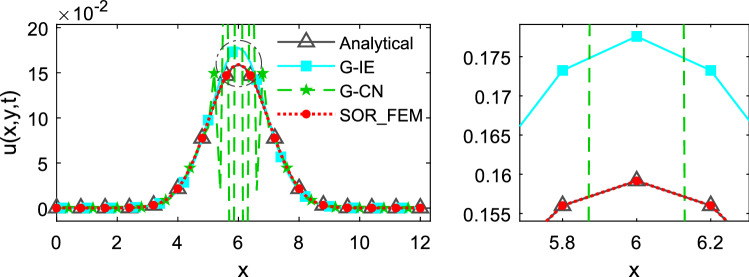
Temperature along the x-axis at time t = 0.1.

**Figure 4:**
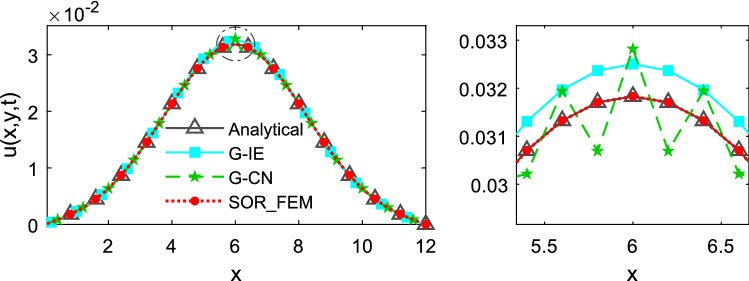
Temperature along the x-axis at time t = 0.5.

**Figure 5:**
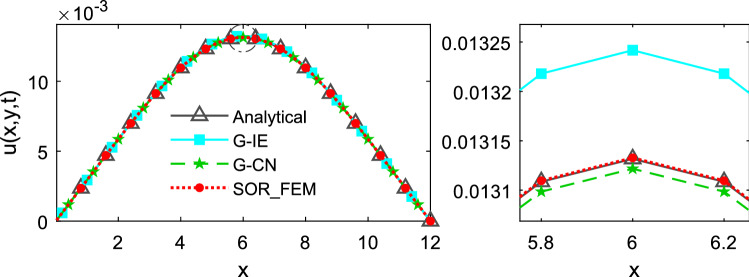
Temperature along the x-axis at time t = 1.2.

Figures 3, 4 and 5 depicts the temperature along the x-axis at different time instants.

**Figure 6:**
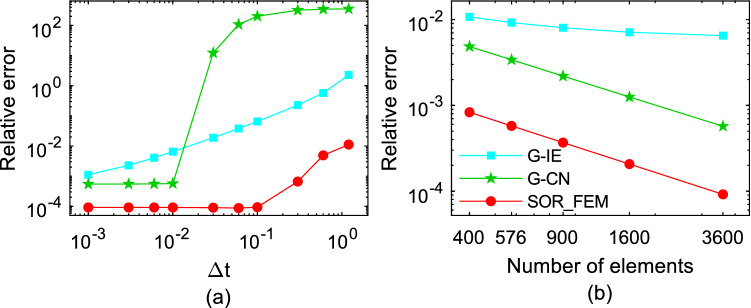
Relative error for different time-step and mesh refinements at time t = 1.2.

The Fig. 6 shows the relative error calculated for different time-steps and different mesh refinements at time $t=1.2$. In Fig. 6a the problem was solved using $\mathrm{\Delta x}=\mathrm{\Delta y}=0.2$ as the element sides. In Fig. 6b the problem was solved using $\Delta t=0.01$ as time-step. The relative error was calculated using the following expression:

32 $$e\left(t\right)=\frac{{\Vert {{\varvec{u}}}_{num}-{{\varvec{u}}}_{an}\Vert }_{2}}{{\Vert {{\varvec{u}}}_{an}\Vert }_{2}},$$

where ${u}_{an}$ is the analytical solution and ${u}_{num}$ is the numerical solution.

Figures 3 and 4 show that in the first time-steps the answer from de G-CN method oscillates and the Fig. 5 shows that after a certain number of time-steps the spurious oscillations are dissipated, that justifies the behavior of the G-CN curve in the Fig. 6a in which the accuracy of the G-CN method is better than the accuracy of the G-IE method for smaller time-steps.

The analysis of the results shows that the proposed method, the SOR_FEM, gives better accuracy in all cases analyzed and with the proposed method is possible to use the Cranck-Nicolson method in time discretization to obtain a numerical solution without spurious oscillation and second order accuracy.

### Example 2

**Figure 7:**
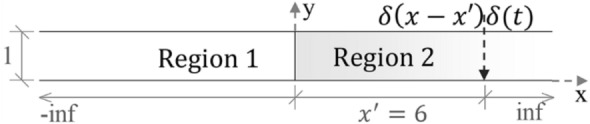
The problem solved in example 2.

In this problem, one finds the temperature resulting from the application of an impulsive line source $\delta \left(x-x{^{\prime}}\right)\delta \left(t\right)$ in an infinite rod, divided into 2 regions, as illustrated in Fig. 7. The physical properties of the model are: thermal conductivity ${\lambda }_{1}=2$ and ${\lambda }_{2}=20$, specific heat capacity ${c}_{1}=0.5$ and ${c}_{2}=2$, mass density ${\rho }_{1}=1$ and ${\rho }_{2}=2$. The initial condition adopted for the two-dimensional FEM model employed is $u(x,y,0)=0$.

According to Carslaw and Jaeger^33^, the analytical solution for this example is given by:

33 $${u}_{an}(x,y,t)=\left\{\begin{array}{ll}\frac{1}{\sqrt{4\pi {\kappa }_{1}t}}{e}^{\frac{{\left(x-{x}{^{\prime}}\right)}^{2}}{4{\kappa }_{1}t}}+\frac{{\lambda }_{1}\sqrt{{\kappa }_{2}}-{\lambda }_{2}\sqrt{{\kappa }_{1}}}{{\lambda }_{1}\sqrt{{\kappa }_{2}}+{\lambda }_{2}\sqrt{{\kappa }_{1}}}\frac{1}{\sqrt{4\pi {\kappa }_{1}t}}{e}^{\frac{{\left(x+{x}{^{\prime}}\right)}^{2}}{4{\kappa }_{1}t}}, & x\ge 0\\ \frac{{\lambda }_{1}{\kappa }_{2}\sqrt{{\kappa }_{1}}}{{\lambda }_{1}\sqrt{{\kappa }_{2}}+{\lambda }_{2}\sqrt{{\kappa }_{1}}}\frac{1}{\sqrt{4\pi {\kappa }_{2}t}}{e}^{\frac{{\left(x-{x}{^{\prime}}\sqrt{{\kappa }_{2}/{\kappa }_{1}}\right)}^{2}}{4{\kappa }_{2}t}},& x<0\end{array}\right.,$$

where $\kappa$ is the thermal diffusivity defined by ${\kappa }_{1}=\frac{{\lambda }_{1}}{{\rho }_{1}{c}_{1}}$ and ${\kappa }_{2}=\frac{{\lambda }_{2}}{{\rho }_{2}{c}_{2}}$ and ${x}{^{\prime}}$ is the source point coordinate, equal to 6 in the present case as shown in Fig. 7.

To apply the developed method, it was used the analytical solution of an infinite homogeneous rod submitted to the same source term illustrated in Fig. 7. According to Carslaw and Jaeger^33^, this analytical solution, denoted as ${u}^{*}$ in Eq. (3), is written as follows:

34 $${u}^{*}=\frac{1}{\sqrt{4\pi {\kappa }_{2}t}}{e}^{-\frac{{\left(x-{x}{^{\prime}}\right)}^{2}}{4{\kappa }_{2}t}}.$$

The time derivative indicated in Eq. (3) is given by:

35 $${\dot{u}}^{*}=\frac{\partial {u}^{*}}{\partial t}=\frac{{\left(x-x{^{\prime}}\right)}^{2}}{8{\kappa }_{2}{t}^{2}\sqrt{\pi {\kappa }_{2}t}}{e}^{-\frac{{\left(x-x{^{\prime}}\right)}^{2}}{4{\kappa }_{2}t}}-\frac{1}{4\sqrt{\pi {\kappa }_{2}{t}^{3}}}{e}^{-\frac{{\left(x-x{^{\prime}}\right)}^{2}}{4{\kappa }_{2}t}}.$$

Physical property of region 2 was chosen for the ${u}^{*}$ solution because it is the region where the source is applied, however this is not mandatory, although the best in this analysis. A good choice is to have physical properties with the same order of magnitude of those of the real problem.

The proposed method (SOR_FEM) employed a uniform mesh composed of 480 quadrilateral bilinear finite elements with four nodes. The element sides were $\Delta x=0.2$ and $\Delta y=0.25$. The time-step used was $\Delta t=0.02$. At the end of each time-step, the proposed method solution was computed by $u=\Delta u+{u}^{*}$.

The SOR_FEM was compared against the methods of the literature given by Eqs. (30) and (31) using the same mesh and time discretization defined above for the proposed method. To represent the effect of the source $\delta (x-x{^{\prime}})\delta (t)$ in Eq. (31) the initial condition $u\left(6,y,0\right)=1/\Delta x$ was adopted. To simulate the infinite medium in all methods analyzed the domain was truncated at the coordinates $x=-12$ and $x=12$ and the analytical solution given by Eq. (33) was used as Dirichlet boundary condition.

**Figure 8:**
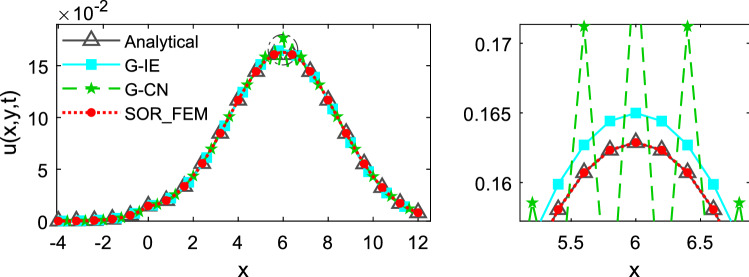
Temperature along the line y = 0.5 at time t = 0.6.

**Figure 9:**
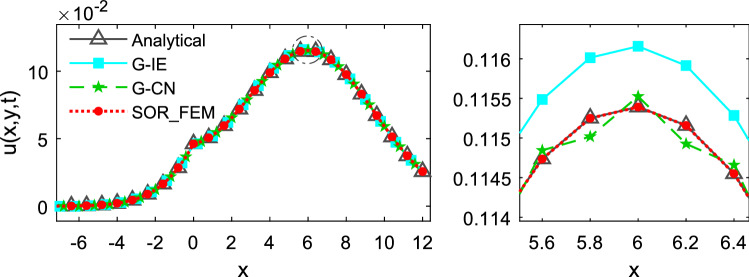
Temperature along the line y = 0.5 at time t = 1.2.

The Figs. 8 and 9 depicts the temperature along the line $y=0.5$ at different time instants.

**Figure 10:**
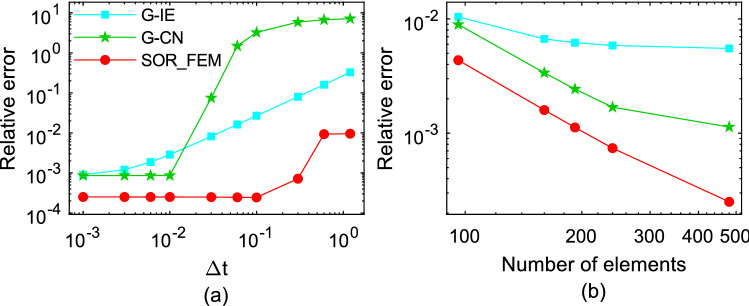
Relative error variation for different time step sizes and mesh refinements at time t = 1.2.

The Fig. 10 shows the relative error calculated for different time-steps and different mesh refinements at time t = 1.2. In Fig. 10a the problems were solved using $\Delta x =0.2$ and $\Delta y =0.25$ as the element sides. In Fig. 10b the problems were solved using $\Delta t=0.02$ as time-step. The relative error was calculated using the Eq. (32).

This analysis illustrates that the SOR_FEM can be applied to non-homogeneous bodies. Like example 1, spurious oscillations were present for early times in the G-CN approach and eliminated by the SOR_FEM approach. Also, the SOR_FEM resulted in better accuracy in all cases analyzed.

It is important to observe that like in the previous application, even for time steps 10 times bigger than the ones employed for G-IE and G-CN the accuracy of the SOR_FEM is still much better.

### Example 3

**Figure 11:**
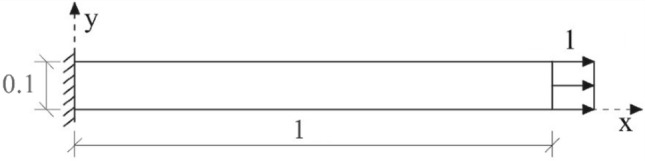
Representation of the problem solved in example 3.

In this example, a two-dimensional finite element space discretization was used to analyze a clamped rod submitted to axial forces instantaneously applied at the initial time, as illustrated in Fig. 11. It is assumed that the entire domain has zero initial displacement, velocity and acceleration. The geometric and physical properties of the model are: thickness $A=0.1$, Young modulus $E={10}^{7}$, mass density $\rho =10$ and Poisson coefficient υ = 0.

The analytical solution for this example is given by:

36 $${u}_{an}\left(x,y,t\right)=\frac{8PL}{{\pi }^{2}EA}\sum_{n=1}^{\infty }\frac{{\left(-1\right)}^{n-1}}{{\left(2n-1\right)}^{2}}sen\left(\frac{2n-1}{2L}\pi x\right)\left[1-\mathrm{cos}\left(\frac{2n-1}{2L}\pi ct\right)\right],$$

where $P$ is the axial force, $L$ is the rod length and $c$ is the wave propagation velocity given by $c=\sqrt{\frac{E}{\rho }}$.

**Figure 12:**
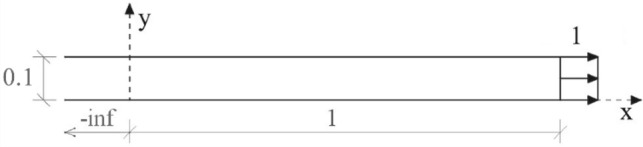
Representation of the semi-infinite media problem used to obtain u*.

To apply the developed method, it is necessary to find the analytical solution of a semi-infinite problem like the problem under analysis, as illustrated in Fig. 12.

The analytical solution ${u}^{*}$ of the semi-infinite problem to be used in Eq. (16) is given by:

37 $${u}^{*}=\frac{P}{EA }\left(ct+x-L\right)\left[1-H\left(L-x-ct\right)\right],$$

where $H$ is the Heaviside function.

The solution given by Eq. (37) is discontinuous, thus it is necessary to smooth its discontinuous part to reduce the spurious oscillations. The following equation is used as a smooth approximation of Eq. (37):

38 $${u}_{s}^{*}=\frac{P}{EA }\left[\frac{\mathrm{ln}\left({e}^{\left(L-ct\right)s}+{e}^{sx}\right)}{s}+ct-L\right],$$

where $s$ is the coefficient that determines the smoothness degree of the response.

**Figure 13:**
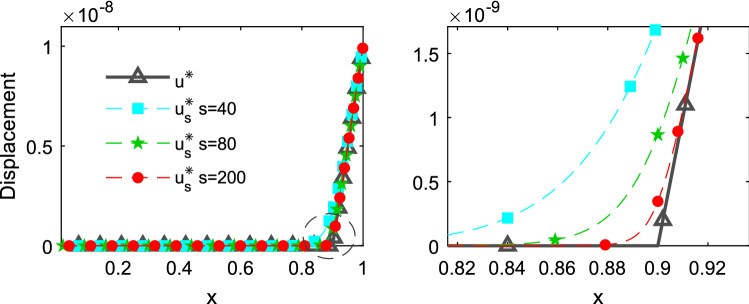
Comparison of ${u}^{*}$ and ${u}_{s}^{*}$ at time t = 0.0001.

The Fig. 13 shows a comparison of the displacements obtained by Eqs. (37) and (38) along the x-axis for different values of s and at time 0.0001. The figure shows that as the value of s increases ${u}_{s}^{*}$ becomes closer to ${u}^{*}$. The value of s should be adopted based on the mesh and time discretization, the finer the mesh and the time discretization the greater the value of s that can be adopted.

Since the rod under analysis is homogeneous the terms ${\varvec{\Delta}}{\varvec{K}}$ and ${\varvec{\Delta}}{\varvec{M}}$ of the Eq. (14) are null, so the SOR_FEM governing equation is given by:

39 $${\varvec{M}}{\varvec{\Delta}}\ddot{{\varvec{u}}}+{\varvec{K}}{\varvec{\Delta}}{\varvec{u}}={\varvec{f}},$$

with Dirichlet boundary condition on the clamped end equal to:

40 $$\Delta u\left(0,y,t\right)=-{u}_{s}^{*}\left(0,t\right),$$

and with Neumann boundary condition equal to:

41 $$\frac{\partial\Delta u\left(x,0,t\right)}{\partial n}=0; \frac{\partial\Delta u\left(x,0.1,t\right)}{\partial n}=0; \frac{\partial\Delta u\left(1,y,t\right)}{\partial n}=0,$$

The proposed method employed a uniform mesh composed of 10,000 quadrilateral bilinear elements with four nodes. The element sides were $\Delta x=0.001$ and $\Delta y=0.01$. The time-step used was ${10}^{-6}$. At the end of each time-step, the proposed method solution was computed by $u=\Delta u+{u}_{s}^{*}$.

As established in the literature, the conventional finite element method results in responses with spurious oscillations when the problem has discontinuous solutions, as in the case of the example under analysis. To illustrate this fact, the Galerkin and Newmark methods were applied to Eq. (14) giving as result the following expression:

42 $$\left(\frac{\gamma }{\beta\Delta {t}^{2}}{\varvec{M}}+{\varvec{K}}\right){{\varvec{u}}}_{{t}_{i}+\Delta t}={{\varvec{f}}}_{{t}_{i}+\Delta t}+{\varvec{M}}\left[\frac{1}{\beta\Delta {t}^{2}}{{\varvec{u}}}_{{t}_{i}}+\left(\frac{1}{\beta\Delta t}\right){\dot{{\varvec{u}}}}_{{t}_{i}}+\left(\frac{1}{2\beta }-1\right){\ddot{{\varvec{u}}}}_{{t}_{i}}\right],$$

The answer obtained through Eq. (42) was called G-NM, this response was found using $\beta =\frac{1}{4}$ and $\gamma =\frac{1}{2}$ and using the same mesh and time discretization described above for the proposed method. The Newmark method was chosen because it is second-order accurate. Since the Newmark method was also applied in time discretization of the SOR_FEM, it is possible to show that the SOR_FEM results in a more stable solution.

**Figure 14:**
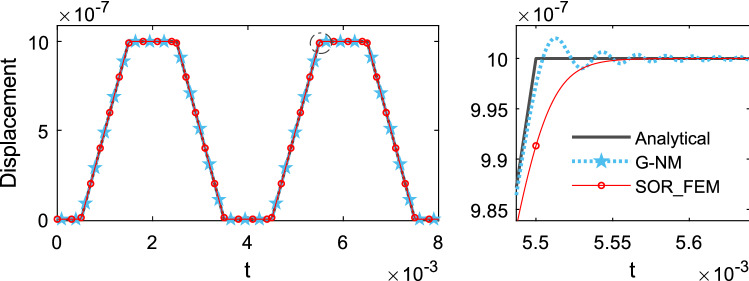
Time history of displacement at the rod center.

**Figure 15:**
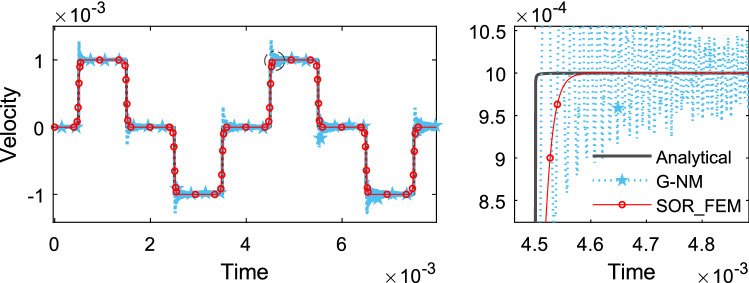
Time history of velocity at the rod center.

**Figure 16:**
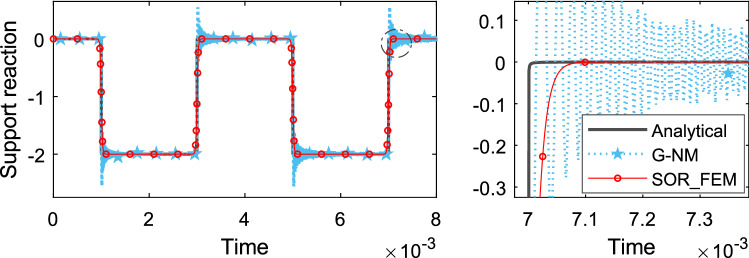
Time history of support reaction.

Figure 14 shows the time history of displacement, Fig. 15 shows the time history of velocity and Fig. 16 shows the time history of support reaction. In Figs. 14 and 15 the response was obtained at the rod center. In all cases the response was found using $s=80$ in the Eq. (38) for the proposed method.

The smooth approximation adopted for the proposed method smoothed the solution around discontinuity points and resulted in good accuracy away from discontinuity points, also it resulted in a more stable solution without spurious oscillations.

**Figure 17:**
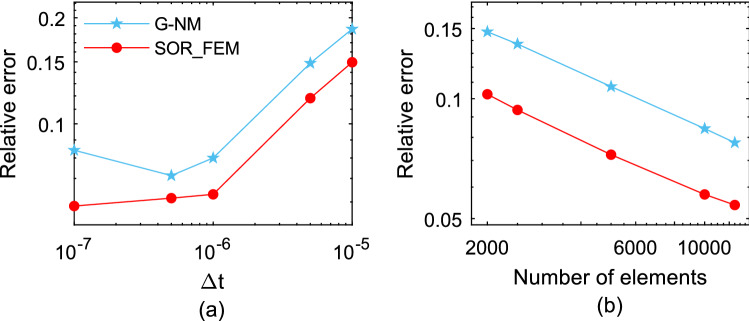
Relative error for velocities for different time step sizes and mesh refinements at time t = 0.005.

The Fig. 17 shows the relative error calculated for different time-steps and different mesh refinements at time t = 0.005. In Fig. 17a the problems were solved using $\Delta x =0.001$ and $\Delta y =0.01$ as the element sides. In Fig. 17b the problems were solved using $\Delta t={10}^{-7}$ as time-step. The relative error, given by Eq. (32), was calculated using the time history of velocity at the rod center. The results show that the proposed method solution converges with the mesh and the time-step refinements. Most important is to notice that the errors of the standard method are much worse than shown in the Fig. 17; as shown in Fig. 15 results in the neighborhood of discontinuities lead to a serious misinterpretation for design.

### Example 4

**Figure 18:**
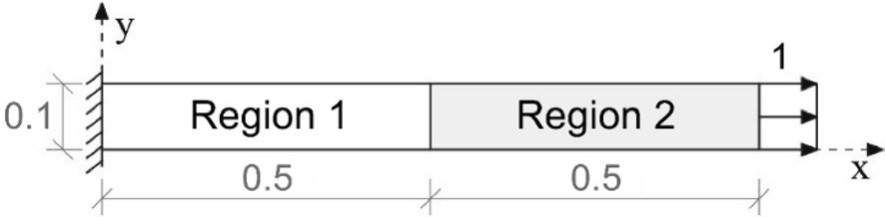
Representation of the problem solved in example 4.

In this example, a two-dimensional finite element space discretization was used to analyze a nonhomogeneous clamped rod submitted to axial forces instantaneously applied at the initial time and divided into two regions, as illustrated in Fig. 18. It is assumed that the entire domain has zero initial displacement, velocity and acceleration. The Young modulus for the region 1 is ${E}_{1}=5\times {10}^{6}$ and the Young modulus for the region 2 is ${E}_{2}={10}^{7}$. The other geometric and physical properties for the two regions are: thickness ${A}_{1}={A}_{2}=0.1$, mass density ${\rho }_{1}={\rho }_{2}=10$ and Poisson coefficient ${\upsilon }_{1}={v}_{2}=0$.

The same analytical solution given by Eq. (38) was used to apply the proposed method in this example. As the mass density is the same for both regions, the ${\varvec{\Delta}}{\varvec{M}}$ term of Eq. (16) is null, so the SOR_FEM governing equation is given by:

43 $${\varvec{M}}{\varvec{\Delta}}\ddot{{\varvec{u}}}+{\varvec{K}}{\varvec{\Delta}}{\varvec{u}}={\varvec{f}}-{\varvec{\Delta}}{\varvec{K}}{{\varvec{u}}}^{\boldsymbol{*}},$$

with boundary conditions given by the Eqs. (40) and (41). The matrices ${{\varvec{K}}}_{0}$ and ${\varvec{\Delta}}{\varvec{K}}$ were determined using ${E}_{0}={E}_{2}$. The same mesh and time discretizations described for example 3 were used.

An analytical expression is not available for this problem, so the proposed method was only compared with the G-NM given by Eq. (42).

**Figure 19:**
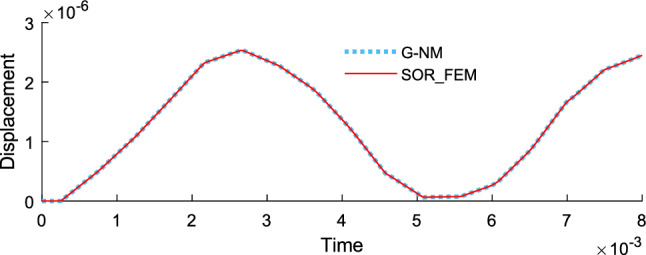
Time history of displacement at the region 2 center.

**Figure 20:**
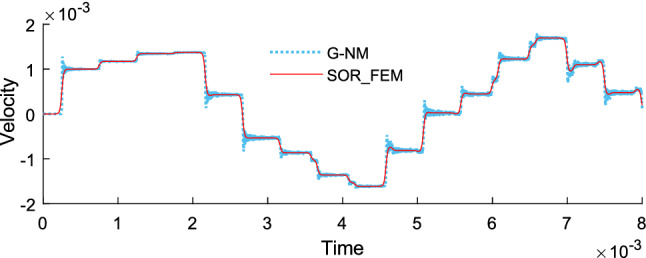
Time history of velocity at the region 2 center.

**Figure 21:**
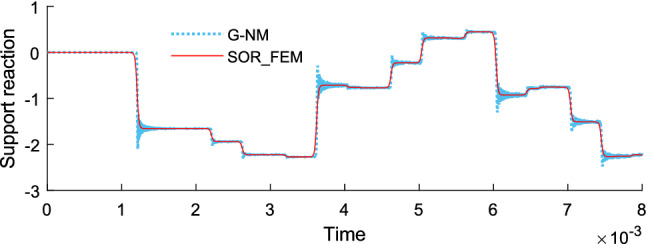
Time history of support reaction.

Figure 19 shows the time history of displacement, Fig. 20 shows the time history of velocity and Fig. 21 shows the time history of support reaction. In Figs. 19 and 20 the response was obtained at the region 2 center point. In all cases the response was found using $s=80$ in the Eq. (38) for the proposed method application. These analyses illustrate that the SOR_FEM can be applied to non-homogeneous bodies. Like example 3, strong spurious oscillations were present in the standard FEM approach, and eliminated by the SOR_FEM approach presented here.

## Conclusion

The results of examples 1 and 2 show that with the SOR_FEM it is possible to obtain numerical solutions of linear heat diffusion problems subjected to concentrate impulsive sources which are accurate and free from spurious oscillations. It is possible to obtain numerical solutions without spurious oscillations applying specific time integration methods, e. g. the Implicit Euler used here eliminated spurious oscillations at the expense of a loss of accuracy for early time, which remained on the neighborhood of the source up to the end of the analysis.

The results of examples 3 and 4 show that with the SOR_FEM it is possible to smooth the response discontinuities of linear wave propagation problems and thus obtain an approximate numerical solution without spurious oscillations. Additionally, the proposed method has the advantage that the formulation is simple to apply, resulting in a finite element matrix form with few additional terms, which has a well-established solution described in the literature.

In the wave propagation problems, the proposed method applications were limited in this paper to cases where it is possible to find a smooth approximation to the analytical solution, ${u}^{*}$, which is used to replace the source term of the problem to be solved, therefore, it is necessary to perform research effort to find techniques capable of smoothing the analytical solutions to be used with the proposed method.

A possible generalization is to employ numerical solutions for homogeneous bodies obtained by series expansions, or any numerical method such as finite or boundary elements, finite differences, finite volumes, etc., instead of analytical ones.

## Data Availability

The data required to reproduce these results are available by contacting the corresponding author.
